# Laboratory-Scale Simulation and Real-Time Tracking of a Microbial Contamination Event and Subsequent Shock-Chlorination in Drinking Water

**DOI:** 10.3389/fmicb.2017.01900

**Published:** 2017-10-04

**Authors:** Michael D. Besmer, Jürg A. Sigrist, Ruben Props, Benjamin Buysschaert, Guannan Mao, Nico Boon, Frederik Hammes

**Affiliations:** ^1^Drinking Water Microbiology Group, Department of Environmental Microbiology, Eawag, Swiss Federal Institute of Aquatic Science and Technology, Dübendorf, Switzerland; ^2^Department of Environmental Systems Science, Institute of Biogeochemistry and Pollutant Dynamics, Zürich, Switzerland; ^3^Center for Microbial Ecology and Technology, Ghent University, Ghent, Belgium; ^4^Key Laboratory of Pollution Processes and Environmental Criteria (Ministry of Education), Tianjin Key Laboratory of Environmental Remediation and Pollution Control, College of Environmental Science and Engineering, Nankai University, Tianjin, China

**Keywords:** continuous real-time flow cytometry, drinking water, bacterial dynamics, disinfection, kinetics

## Abstract

Rapid contamination of drinking water in distribution and storage systems can occur due to pressure drop, backflow, cross-connections, accidents, and bio-terrorism. Small volumes of a concentrated contaminant (e.g., wastewater) can contaminate large volumes of water in a very short time with potentially severe negative health impacts. The technical limitations of conventional, cultivation-based microbial detection methods neither allow for timely detection of such contaminations, nor for the real-time monitoring of subsequent emergency remediation measures (e.g., shock-chlorination). Here we applied a newly developed continuous, ultra high-frequency flow cytometry approach to track a rapid pollution event and subsequent disinfection of drinking water in an 80-min laboratory scale simulation. We quantified total (TCC) and intact (ICC) cell concentrations as well as flow cytometric fingerprints in parallel in real-time with two different staining methods. The ingress of wastewater was detectable almost immediately (i.e., after 0.6% volume change), significantly changing TCC, ICC, and the flow cytometric fingerprint. Shock chlorination was rapid and detected in real time, causing membrane damage in the vast majority of bacteria (i.e., drop of ICC from more than 380 cells μl^-1^ to less than 30 cells μl^-1^ within 4 min). Both of these effects as well as the final wash-in of fresh tap water followed calculated predictions well. Detailed and highly quantitative tracking of microbial dynamics at very short time scales and for different characteristics (e.g., concentration, membrane integrity) is feasible. This opens up multiple possibilities for targeted investigation of a myriad of bacterial short-term dynamics (e.g., disinfection, growth, detachment, operational changes) both in laboratory-scale research and full-scale system investigations in practice.

## Introduction

Contamination events occasionally occur in different parts of drinking water treatment, storage, and distribution systems, with potentially dire health consequences as a result (Craun et al., 1998; Craun and Calderon, 2001; LeChevallier et al., 2003; Nygård et al., 2006; Breitenmoser et al., 2011). Apart from treatment failure (e.g., ozone, chlorine and/or membrane failure), problems in the distribution system such as low pressure, backflow, and unintentional cross-connections of water with lower quality such as wastewater or gray water are a major source of such contamination events (Antoun et al., 1999; Craun and Calderon, 2001; Hrudey et al., 2003; LeChevallier et al., 2003; Westrell et al., 2003; Besner et al., 2007; Pontius and Evans, 2008; Laine et al., 2011). Evidently, contamination events can occur in different network locations with different contaminant types and concentrations (United States Environmental Protection Agency [EPA], 2001; Hrudey and Hrudey, 2007; Moreira and Bondelind, 2017). The contamination often occurs rapidly since small volumes of polluted water can contaminate very large volumes of drinking water (Besner et al., 2001, 2011; Westrell et al., 2003). Several seminal review papers have documented the causes, pathogenic agents, and epidemiologic consequences of recent drinking water contamination events in industrialized countries (Hrudey and Hrudey, 2007; Moreira and Bondelind, 2017). The potential severity of negative health impacts and the rapid and transient nature of such contamination events call for rapid and sensitive detection methods to automatically monitor critical control points in drinking water systems (United States Environmental Protection Agency [EPA], 2001; Brown and Hussain, 2003; Allen et al., 2015).

A variety of methods and sensors are used to detect contamination events such as wastewater entering drinking water systems, including fluorescence based monitoring, sensors for (residual) chlorine, dissolved oxygen, particle counters, turbidity, and spectral absorption (Skadsen et al., 2008; Hambly et al., 2010; Gosselin et al., 2013; Allen et al., 2015; United States Environmental Protection Agency [EPA], 2015; Page et al., 2017). Abiotic sensors can give fast warnings when a change in general water quality occurs (Gosselin et al., 2013; United States Environmental Protection Agency [EPA], 2015; Page et al., 2017). However, *direct* measurements for microbial water quality are still predominantly cultivation based (Brown and Hussain, 2003; Allen et al., 2015). If a contamination event is suspected (for example based on abiotic measurements or consumer complaints), water utilities need to collect water samples, process them in the laboratory, and usually wait at least 24 h for the results. This procedure is very time consuming and labor intensive – especially if large systems are affected. The time-to-results is extremely long considering the implications for consumers (e.g., water boiling orders, switch to bottled water) and severely limits the water utility with respect to solving the problem (e.g., localizing the source of contamination, testing the success of corrective measures) (Krewski et al., 2002; United States Environmental Protection Agency [EPA], 2015). Moreover, even though the vast majority of waterborne disease outbreaks are caused by protozoa (e.g., *Cryptosporidium*, *Giardia*) and viruses (e.g., Norovirus, Rotavirus) (Moreira and Bondelind, 2017), conventional monitoring for indicator bacteria remains the first level of screening for many water utilities (Allen et al., 2015).

To effectively and directly monitor rapid contaminations (Brown and Hussain, 2003) but also subsequent remediation procedures such as disinfection through shock-chlorination (Miettinen et al., 2001; Van Nevel et al., 2017a), microbial monitoring tools with very high temporal resolution are needed. This is only feasible through full automation of *in situ* sampling, sample preparation, and measurements. Rapid microbial detection methods that are suitable to assess both contamination and disinfection include for example ATP assays (Vang et al., 2014; Nescerecka et al., 2016) and flow cytometry (FCM) using viability staining protocols (Hammes et al., 2012; Helmi et al., 2015). With respect to FCM, examples of automated, *in situ* monitoring of microbial dynamics were previously shown for total cell concentration (TCC) and discrete sampling (15-min resolution) (Brognaux et al., 2013; Besmer et al., 2014, 2016; Besmer and Hammes, 2016) and for enzymatic reactions as well as GFP-labeled bacteria and continuous sampling (1-min resolution) (Nolan and Sklar, 1998; Broger et al., 2011; Arnoldini et al., 2013). TCC measurements, based on a single stain protocol, are suitable to detect changes in bacterial concentrations, which the inflow of wastewater, for example, would cause (Prest et al., 2013). To assess the effect of oxidative disinfection on bacteria, i.e., primarily cell membrane damage, a viability staining protocol can be applied to determine for example the intact cell concentration (ICC) (Phe et al., 2005; Berney et al., 2007; Ramseier et al., 2011; Mustapha et al., 2015; Lee et al., 2016). Ideally, both measurements (TCC and ICC) are performed in parallel to maximize information gain (Van Nevel et al., 2017b).

The specific goals of this study were: (1) to monitor the effect of a rapid contamination event and subsequent shock-chlorination remediation response on bacterial TCC and ICC in a simplified simulation of a drinking water reservoir; and (2) to demonstrate the exciting possibilities offered by parallel FCM measurements of TCC and ICC in real-time at very high temporal resolution. The novelty of this study is the use of ultra-high frequency microbial monitoring of a laboratory scale simulation of a drinking water contamination event and subsequent emergency measures (i.e., chemical disinfection).

## Materials and Methods

### Experimental Setup

**FIGURE 1:**
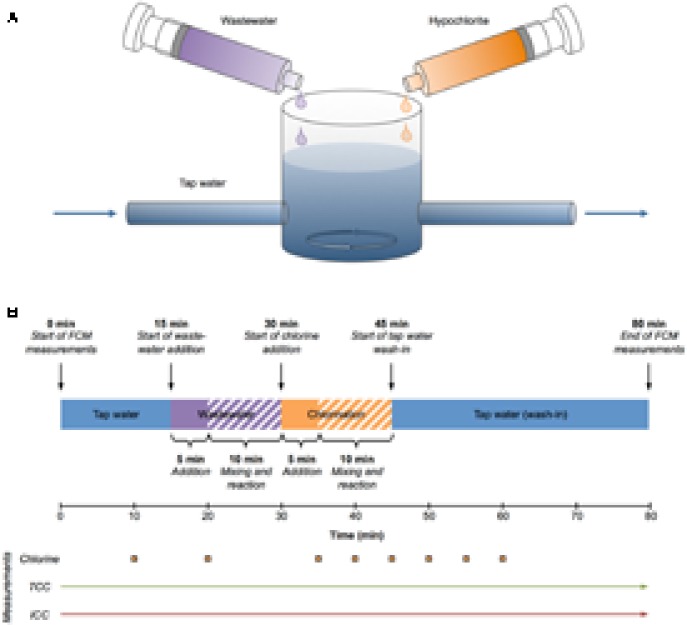
Experimental setup **(A)** and sequence **(B)**. The setup included a stirred batch/flow-through reactor fed with tap water, a syringe with wastewater, and a syringe with chlorine solution (both operated with syringe pumps) **(A)**. The tap water was pumped with two peristaltic pumps (not displayed) and solution addition from the syringes was controlled by syringe pumps (not displayed). The experimental sequence **(B)** consisted of (1) 15 min of tap water (batch mode), (2) wastewater addition for 5 min (solid purple bar) and 10 more minutes for mixing and reaction (shaded purple bar), (3) chlorine addition for 5 min (solid orange bar) and 10 more minutes for mixing and reaction (shaded orange bar), and (4) wash-in of tap water (flow-through mode) for 35 min. Chlorine measurements were done 10, 20, 35, 40, 45, 50, 55, and 60 min after the start of the experiment (orange squares, bottom). Total (TCC) and intact (ICC) cell concentrations were measured continuously.

A drinking water reservoir was simulated with a 600 ml glass reactor initially filled with 500 ml of non-chlorinated tap water and continuously mixed with a magnetic stirrer (**Figure 1A**). The tap water characteristics were: pH = 7.9, TOC = 0.8 mg l^-1^, conductivity = 461 μS cm^-2^ and chlorine = 0 mg l^-1^. This setup was started as a stirred batch reactor and later operated as a stirred flow-through reactor (**Figure 1B**). In the latter case, fresh, non-chlorinated tap water was pumped into the reactor and the mixed water was pumped out of the reactor with two peristaltic pumps, maintaining a constant water level in the reactor. **Figure 1B** gives an overview of the experimental sequence. After 15 min of tap water baseline measurements, raw municipal wastewater (10-fold pre-diluted with tap water) was added with a syringe pump at 3 ml min^-1^ for 5 min to simulate a contamination event. Ten minutes later (30 min after the start of the experiment) the chlorine (hypochlorite, 72 mg l^-1^ free chlorine) addition with a syringe pump was started at 2.5 ml min^-1^ for 5 min to reach a final concentration of about 1.8 mg l^-1^ free chlorine in the reactor, simulating emergency remediation procedures. After another 10 min (45 min after the start of the experiment), the system was switched to flow-through mode and fresh tap water was pumped through the glass reactor at a rate of 100 ml min^-1^ for 35 min. The experiment was terminated after 80 min.

### Continuous Real-Time Flow Cytometry

Two identical, custom-made automated sampling, staining, and incubation modules were combined with an Accuri C6 flow cytometer (BD Accuri, San Jose, CA, United States) each. The two flow cytometers were calibrated with calibration beads before the experiment. The automation modules were in principle similar to the systems described in (Hammes et al., 2012), but operated continuously rather than at discrete intervals. Water samples were drawn continuously from the reactor at 0.3 ml min^-1^ by a high-precision pump. For TCC measurements, the sample was mixed continuously with SYBR Green I (SG) (final concentration: 1:10,000; rate: 0.3 ml min^-1^) in a mixing chamber. For ICC measurements, the sample was mixed continuously with a combination of SG and propidium iodide (PI) [final concentration: 1:10,000 (SG) and 6 μM (PI); rate: 0.3 ml min^-1^]. In order to avoid reaction between chlorine on the fluorescent stains, both staining solutions also contained sodium nitrite (final concentration: 0.8 mM). The quenched and stained sample streams were incubated for 10 min at 37°C in a continuously flowing incubation loop and then directed to the flow cytometer at 0.6 ml min^-1^, where they were measured continuously at 100 ms resolution [flow rate: 14 μL min^-1^; mode: “unlimited run”; lower threshold on the green fluorescence (FL1-H): 1,000, also see Arnoldini et al. (2013) and Heck et al. (2014)].

### Chlorine Measurements

Chlorine measurements were performed on grab samples taken 10, 20, 35, 40, 45, 50, 55, and 60 min after the start of the experiment (**Figure 1B**). The chlorine concentrations were measured with a *N*,*N*-diethyl-*p*-phenylenediamine (DPD) colorimetric kit (Hach-Lange LCK 310, Germany) and a bench top spectrophotometer (Hach-Lange DR3900, Germany). After reading a blank sample, water samples were added to reagent-containing cuvettes and incubated for 1 min. The resulting intensity of red color was proportional to the chlorine concentration. The kit has a linear range of 0.05–2 mg l^-1^ and an accuracy of ±3%.

### Data Analysis

Fixed gates as defined by Prest et al. (2013) were used to separate bacteria from background signals and to distinguish between high (HNA) and low (LNA) nucleic acid content bacteria (i.e., flow cytometric fingerprints). Gated FCM measurements were exported to csv files at 100 ms resolution and all individual recorded events (i.e., bacterial cells) were allocated to bins of 60 s duration based on their time tags [see Supplementary Table S1 for an example and Arnoldini et al. (2013)].

### Predicting TCC Values

The expected TCC was calculated for the four basic phases of the experiment [i.e., (1) tap water, (2) addition of wastewater, (3) addition of chlorine, and (4) wash-in of fresh tap water (**Figure 1B**)] at discrete 1-min resolution. The initial tap water phase was represented by the measured average TCC (1–15 min). The effect of wastewater addition was calculated based on offline TCC measurements of the pre-diluted wastewater (14,700 cells μl^-1^) prior to the experiment and the addition rate of 3 ml min^-1^ assuming perfect mixing (16–25 min). No effect of chlorine on TCC was assumed. The wash-out of the contamination by the wash-in of fresh tap water was calculated based on the tap water average TCC, the size of the reactor (500 ml), and the flow rate of tap water (100 ml min^-1^) resulting in a dilution rate of 0.2 min^-1^ assuming perfect mixing (46–80 min).

## Results

### Simplified Simulation of Drinking Water Contamination and Remediation

**FIGURE 2:**
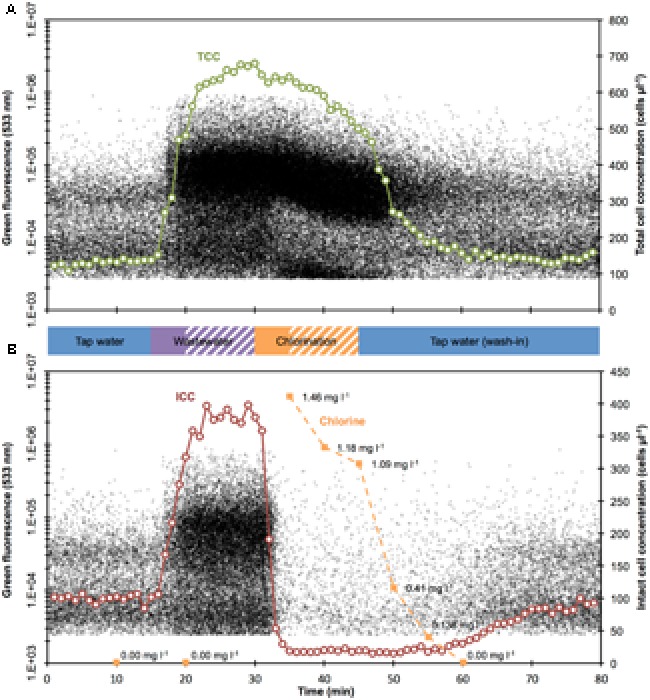
Combined density plots and cell concentration values for TCC (**A**, green) and ICC (**B**, red) over time. Each black dot represents a bacterium. The cell concentrations are 1-min cumulative values of bacteria. Both wastewater and chlorine were added for 5 min (solid bars) before 10 more minutes were allowed for mixing and reaction (shaded bars), respectively. Chlorine measurements (**B**, orange) were below detection limit for the first two measurements (before chlorine addition) and for the last measurements.

Real-time TCC and ICC measurements were taken continuously and in parallel throughout the entire experiment until termination after 80 min (**Figure 1B**). The experiment included four basic phases (**Figure 1B**). The first 15 min were baseline measurements of non-chlorinated tap water. This was followed by the addition of diluted wastewater during 5 min followed by a 10-min time window of mixing and reaction. Thereafter, chlorine was added during 5 min given an additional 10 more minutes to react. Subsequently, 45 min after experiment initiation, fresh (non-chlorinated) tap water was flushed in and the reactor operation was switched from batch mode to flow-through mode. The continuous FCM data (**Figure 2**) was collected with a 10 min delay (for staining and incubation). For the purpose of direct comparison, the measured data was aligned with the sampling times (**Figures 1, 2**).

### Wastewater Contamination Increases Total and Intact Cell Concentrations

**FIGURE 3:**
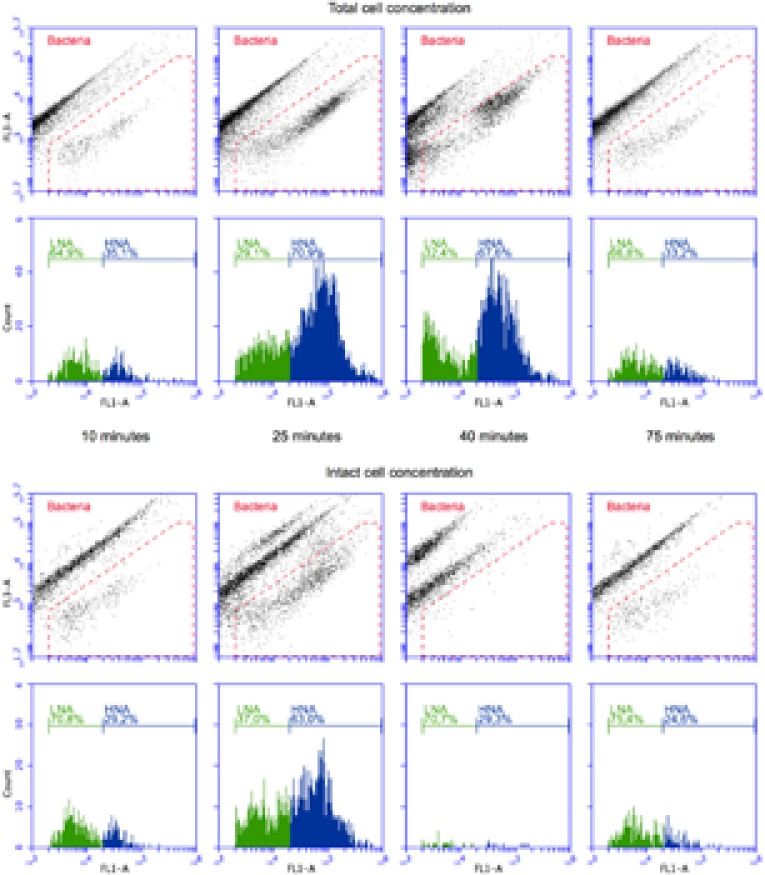
Density plots and histograms for TCC (SG staining, **top**) and ICC (SG PI staining, **bottom**) measurements of selected 1 min windows at different stages of the experiment: (i) tap water (10 min), (ii) wastewater contamination (25 min), (iii) chlorination effect (40 min), and (iv) return to tap water (75 min). The green fluorescence values (*x*-axis) correspond to the data in **Figure 2** but show the red fluorescence (*y*-axis) in addition. The histograms only contain the data within the bacterial gate (red). Distinction between LNA and HNA bacteria follows the definition of Prest et al. (2013).

The experiment started with non-chlorinated tap water, resulting in a period of stable measurements (TCC: 130.6 ± 8.3 cells μl^-1^; ICC: 99.6 ± 5.9 cells μl^-1^; *n* = 15; **Figure 2**). Such stable measurements are expected from the experimental set-up (closed reactor with continuous stirring), and demonstrate the stable performance of the continuous staining prototype. Spiking of diluted wastewater in the reactor (start after 15 min) resulted in a rapid increase in both TCC and ICC. Concentrations of 600 cells μl^-1^ (TCC) and 380 cells μl^-1^ (ICC) were exceeded 8 min later (23 min after the start of the experiment) (**Figure 2**). While TCC continued to increase to a maximum of 679.4 cells μl^-1^ (fivefold increase from tap water; 30 min after the start of the experiment), ICC was rather stable for 8 min (383.3 ± 10.6 cells μl^-1^; fourfold increase from tap water). The simulated ingress of wastewater influenced not only the concentration but also the composition of bacteria, indicated by the flow cytometric fingerprint. In **Figure 3**, the substantial changes of the ratios between (small) LNA and (large) HNA content bacteria can be seen both for TCC and ICC. As can be seen in the histograms (**Figure 3**), the tap water after 10 min contained 65% LNA content bacteria whereas the contaminated mixture after 25 min contained only 29%. For ICC, the percentage of LNA content bacteria decreased from 71% (tap water) to 37% (contaminated mixture). The corresponding density plots in **Figure 3** show that this shift was due to an over-proportional increase of HNA content bacteria. This is in line with the high percentage of HNA content bacteria present in the raw wastewater (61.3 ± 1.1%). Chlorine concentrations were below the detection limit (0.05 mg l^-1^) at this stage, since the tap water used for the experiment is distributed without residual chlorine and the wastewater neither contained chlorine (**Figure 2B**).

### Chlorination Damage Is Rapid While Washout Is Gradual

The simulated shock-chlorination (initial calculated concentration: 1.8 mg l^-1^) was triggered 30 min after the start of the experiment and was detectable within 3 min of addition (33 min after the start of the experiment) when both ICC and TCC started to decrease. ICC, reflecting the cell membrane permeabilization caused by chlorine, dropped rapidly below 30 cells μl^-1^ 1 min later (34 min after the start of the experiment) where it remained for 25 min (19.6 ± 3.7 cells μl^-1^, i.e., approximately 95% reduction). Interestingly, TCC also decreased during chlorination, albeit at a clearly slower rate of 10.6 cells μl^-1^ min^-1^ for 16 min (minutes 31 – 46 after the start of the experiment). The measured free chlorine concentration, following addition of the full volume of chlorine (35 min after the start of the experiment), was 1.46 mg l^-1^, suggesting considerable reaction with the bacteria and water matrix. Chlorine consumption was 0.34 mg l^-1^ in the first 5 min following addition and was responsible for all of the observed cell damage (95% in ICC reduction in the same period). The chlorine concentrations further declined to 1.09 mg l^-1^ within 10 min (45 min after the experimental start). The addition of chlorine had very little influence on the flow cytometric fingerprint. For TCC, the corresponding density plot in **Figure 3** (after 40 min) shows some effect (i.e., shifts of the clusters) but this did not translate into large changes in the percentage of LNA content bacteria (slight increase from 29 to 32%). For ICC, meaningful determination of the fingerprint was not feasible given the low number of events within the bacterial gate in the density plot after 40 min (**Figure 3**).

Exactly 45 min after the experimental start, the wash-out of the contamination was initiated by switching the reactor to flow through mode with fresh non-chlorinated tap water being washed in. TCC responded within 2 min (47 min after the start of the experiment) and decreased rapidly following an exponential decrease to initial tap water levels of approximately 140 cells μl^-1^. Similarly but later (59 min after the start of the experiment), ICC started to return to initial levels of tap water of approximately 95 cells μl^-1^. After the wash-in of the tap water (starting 45 min after the start of the experiment), both TCC and ICC fingerprints returned to initial values as can be seen in both the density plots and histograms after 75 min in **Figure 3**. The chlorine concentration dropped rapidly to 0.138 mg l^-1^ within 10 min (55 min after the start of the experiment) after the wash-in of tap water was started (**Figure 2B**). Sixty minutes after the start of the experiment, the values were below the limit of detection.

### TCC Measurements Match Predictions of Simulated Data

**FIGURE 4:**
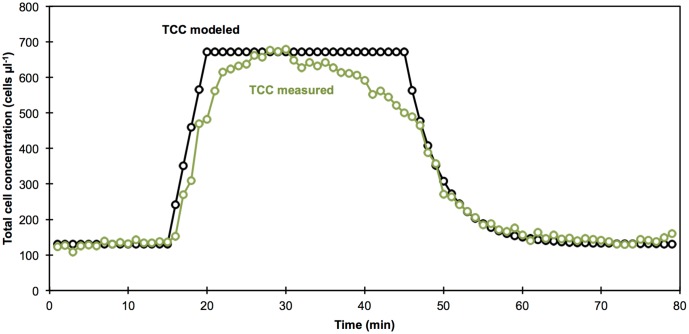
Comparison of measured (green circles) and calculated (black circles) TCC over the course of the experiment. From left to right: (1) the stable tap water phase was assumed based on the measured average TCC (until 15 min), (2) the addition of wastewater was calculated based on pre-measurement of TCC in the wastewater and the addition rate (16–25 min), (3) the observed decrease of TCC after chlorination (26–45 min) was not predicted by the calculated data, (4) the washout was calculated based on the known TCC of the inflowing tap water, the size of the reactor, and the flow rate of tap water (46–80 min).

A straightforward data simulation, based on the measured TCC of the tap water and wastewater and addition rates (see section Experimental Setup), allowed for a prediction of the TCC in the experiment over time (**Figure 4**). The TCC measurements mostly resembled the simulated data (**Figure 4**). The measured temporal evolution of TCC during the addition of wastewater was almost identical with the predicted values. The predicted plateau at high TCC between the addition of wastewater and chlorine was reached but not stable. Interestingly, an approximately linear decrease in TCC was observed in the measurements after the addition of chlorine. Finally, the measurements for the wash-out of the contamination at the end of the experiment were in strong agreement with the predicted data. The perceived time shift (i.e., “delay”) of the measured TCC of approximately 1 min lies within the temporal resolution of the detection method and will also partly be caused by mixing and technical limitations of the reactor setup. Nevertheless, the good correlation with the simulated data demonstrates that the continuous staining, incubation and measurements done here were capable of delivering high quality data over an extended measurement period.

## Discussion

The purpose of this study was to demonstrate the application potential of a novel continuous FCM approach to track dynamic microbial events as changes in TCC and ICC in real time. Such events have been tracked over extended time scales at low temporal resolution with established technologies (Besmer et al., 2014; Page et al., 2017), but to date not at the high temporal frequencies demonstrated here. To this end, we constructed a laboratory-scale experimental set-up enabling simplified event simulation. While the set-up and event simulation were within the framework of realistic conditions (i.e., real drinking water, real wastewater, typical chlorine concentrations), the purpose was not *per se* a direct simulation of any known/specific contamination event, which can vary considerably in complexity and nature.

### Rapid Microbial Changes Were Tracked in Detail, Quantified, and Differentiated

Rapid changes in bacterial concentration, composition, and viability due to: (1) a simulated contamination of drinking water (increase in concentration), (2) the effect of chemical disinfection (decrease in concentration due to cell damage), and (3) the wash-out of both the contamination and the chlorine by flushing of the reactor with fresh tap water, were monitored in real-time at very high temporal resolution.

The effect of contamination was detectable 1 min after the addition started (**Figure 2**). Assuming perfect mixing, this was equivalent to a 0.6% (v/v) share in the reactor of the 10-fold pre-diluted wastewater. This would be equivalent to 60 l of undiluted wastewater entering into a drinking water reservoir with a volume of 100 m^3^. Our experiment was a very basic simulation of a single, high-load contamination event in a static reservoir. Due to the high TCC in the wastewater (more than 100-fold higher than drinking water), at this point already more than 70% of all bacteria in the mixed water originated from wastewater. This is in accordance with contamination levels observed in karstic groundwater following precipitation (Page et al., 2017) and with the wastewater contamination volumes reported for the infamous outbreak in Nokia, Finland (Laine et al., 2011). Real-world contamination scenarios can be considerably more complex than a laboratory-scale simulation, such as a small leak with varying loads into a continuously flowing distribution system over and extended time period (Hrudey and Hrudey, 2007; Moreira and Bondelind, 2017). However, Prest et al. (2013) reported that drinking water contamination could be detected for as little as 4% of contaminating bacteria by statistical analysis of flow cytometric fingerprints (combination of percentage HNA content bacteria and TCC). More advanced fingerprinting approaches potentially allow for even more sensitive detection of contamination and other changes (Koch et al., 2013, 2014; Props et al., 2016). Such a fingerprinting example for the ICC data set of this experiment can be found in Supplementary Figure S1. Until now, the automated technology used here has only been demonstrated once before to track smaller fluctuations (between 100 and 300 cells μL^-1^) in a drinking water treatment plant for TCC only (Besmer and Hammes, 2016). The high sensitivity and the demonstrated automation of continuous *in situ* measurements suggest considerable potential for this technology to be further assessed as a direct microbial sensor in early-warning systems.

Shock-chlorination is often used as emergency response to (suspected) contamination events (Kukkula et al., 1999; Hafliger et al., 2000; Laine et al., 2011; Van Nevel et al., 2017a). The oxidative effect of the added chlorine on suspended bacteria became visible almost immediately in ICC, where the concentration dropped tenfold within 4 min (**Figure 2B**). This is in line with the ATP-based viability assessment of Nescerecka et al. (2016) that found complete disinfection of *Escherichia coli* pure cultures and river water bacteria by chlorine after 5 min for (initial) chlorine concentrations above 0.70 mg l^-1^. Similarly, Ramseier et al. (2011) measured ICC below 30 cells μl^-1^ (equivalent to approximately 20% ICC and an 80%-reduction) in tap water for comparable chlorine exposures as applied in our experiment (i.e., approximately 10 mg min l^-1^). Earlier, Lisle et al. (1999) reported more than 90% injured cells based on plate counts after 5 min and an initial chlorine concentration of 0.50 mg l^-1^. Even at low chlorine concentrations of 0.14 mg l^-1^ and after 10 min of inflowing fresh tap water, the ICC remained very low (**Figure 2B**). Similarly low ICC values were reported by Gillespie et al. (2014) for low residual chlorine concentrations in distribution networks. In our study, ICC only started to increase after approximately three volume changes with fresh, non-chlorinated tap water in the reactor and chlorine concentrations below the limit of detection of the used detection kit. It is well known that bacteria are more sensitive to chlorine disinfection than viruses (Payment, 1999), while the latter are often the primary agents of disease in waterborne outbreaks (Hrudey and Hrudey, 2007; Moreira and Bondelind, 2017). Our data do not suggest that ICC data should replace specific detection of pathogens during waterborne outbreaks, but that real-time ICC data can provide operators with first information on the effectiveness of a shock-chlorination treatment, similar to recent data shown by Van Nevel et al. (2017a) following network maintenance operations.

Chlorination also led to a moderate but clear linear decrease in TCC (**Figure 2A**) and a subtle but distinguishable shift of the bacterial clusters in the FCM density plots (**Figure 3**). We attribute this to increasing cell damage on DNA level that occurs in addition to the membrane damage that is detected with the combined SG and PI staining. As Phe et al. (2005) and Lee et al. (2016) suggested for investigations on ozonation and chlorination, respectively, increased DNA damage can reduce the binding of nucleic acid stains and thus lower the fluorescence signals. The observed slight increase of percentage LNA content bacteria in TCC during chlorination resulted from a decrease in HNA content bacteria. This implies that HNA content bacteria are more susceptible to the above-mentioned DNA damage through chlorination. This would be in line with the observation of Ramseier et al. (2011) that HNA content bacteria suffered faster damage from chlorination than LNA content bacteria, and reported community shifts during disinfection with chloramine Chiao et al. (2014), suggesting varying susceptibility of different groups of bacteria to disinfection.

### Implications, Applications, and Challenges

The present study was carried out on laboratory scale, with the purpose of demonstrating the potential application of continuous real-time monitoring to track events. Nonetheless, the relevance of such scenarios for real drinking water systems in industrialized countries is evident. Examples of contamination of drinking water wells, reservoirs, and distribution systems by wastewater and manure (sometimes coupled to insufficient chlorination) include cases from Finland, Canada, Norway, and Switzerland (Hafliger et al., 2000; Miettinen et al., 2001; Brown and Hussain, 2003; Hrudey et al., 2003; Nygård et al., 2006; Breitenmoser et al., 2011; Laine et al., 2011; Montandon et al., 2016). The experimental set-up in the present study served a demonstration purpose and does not nearly present all the challenges of real-world situations. For example, bacterial concentrations in treated drinking water do not necessarily remain stable, showing both operational fluctuations (e.g., Besmer and Hammes, 2016; Besmer et al., 2016) and seasonal fluctuations (Prest et al., 2016). It is essential that such dynamics (so-called fluctuating baselines) are quantified and understood in order to distinguish contamination events from natural fluctuations. Also, contamination events can occur in different locations within a drinking water network (Hrudey and Hrudey, 2007; Moreira and Bondelind, 2017), thus severely challenging operators with respect to the best locations to install online monitoring equipment.

The data shown here demonstrate first steps toward establishing tools to directly and continuously monitor rapid changes in bacterial concentrations and viability in water. In contrast to previously published discrete, online FCM systems (Brognaux et al., 2013; Besmer et al., 2014, 2016; Besmer and Hammes, 2016), the prototype used here drew, stained, and processed samples continuously, and the temporal resolution after data analysis was considerably higher (1 min rather than 15 min). Based on data acquisition at 100 ms resolution and preliminary statistical assessment of the binning (data not shown), the temporal resolution may even be lowered to approximately 10 s (especially for high bacterial concentrations). Optimization of staining and incubation is feasible (Prest et al., 2013) and thus the current time-to-results of 10 min may be lowered further where needed. As such, this technology represents a breakthrough in FCM automation. However, the approach as presented herein measures general microbial variables, namely total and intact cell concentrations (TCC and ICC), which are not necessarily reflective of changes in conventional variables such as indicator organisms (e.g., *E. coli*) or reflective of specific pathogenic organisms (e.g., *Cryptosporidium*). Moreover, FCM measures specifically suspended bacterial cells as individual events, and therefore clusters (e.g., biofilm clumps sloughed during disinfection and/or flushing, would be recorded as individual particles/cells, hence underestimating actual microbial loads in a system (discussed in detail in Van Nevel et al., 2017b). Hence, additional research is needed in real-case scenarios in order to support an operator’s decision to implement emergency response measures in the case of observed changes in TCC or ICC.

On a more fundamental level, this experiment demonstrated for the first time the potential of continuous, parallel staining and analysis of both TCC and ICC. This allowed us to track the effect of oxidation on bacterial viability *in situ* and in real-time, which complements the work of Arnoldini et al. (2013) on the time-resolved effects of oxidation of algae with auto-fluorescence measurements. Such high-frequency measurements have considerable possibilities in basic laboratory-scale research. Potential further investigations include real-time tracking of various different disinfection strategies on bacteria (e.g., heat, surfactants, sunlight, heavy metals, membrane targeting antibiotics) (Suller and Lloyd, 1999; Berney et al., 2006; Bigoni et al., 2014). The differentiation between TCC and ICC is a strength of FCM (Helmi et al., 2015; Van Nevel et al., 2017b) and can easily be extended to other viability/activity dyes to enable deeper insights into disinfection processes (Nebe-von-Caron et al., 2000; Tracy et al., 2010). While manual and automated ATP-assays can also deliver information on viability (Vang et al., 2014; Nescerecka et al., 2016), continuous ATP measurements are currently not possible. Moreover, several existing and emerging drinking water monitoring tools are either not differentiating bacteria from abiotic particles (e.g., Gosselin et al., 2013; Page et al., 2017) or cannot make the differentiation between viable and non-viable bacteria (Lopez-Roldan et al., 2013; Hojris et al., 2016).

The application presented here shows promise and calls for further testing in laboratory-, pilot-, and full-scale systems. Potential investigations include (1) measurements of worst-case contamination scenarios under realistic conditions (Westrell et al., 2003), (2) real time testing of the influence of operational changes (e.g., changes in flow rates, backwashing of filters) on bacterial concentrations in drinking and process water systems (Hoefel et al., 2005; Besmer and Hammes, 2016), (3) assessment of value for integrated early warning systems based on a combination of direct microbial measurement tools at critical control points (Janke et al., 2006; Murray et al., 2010; Storey et al., 2011; United States Environmental Protection Agency [EPA], 2015), (4) dynamic, real-time investigation of (dosage-dependent) disinfection and other kinetics (Hoefel et al., 2005; Phe et al., 2005; Ramseier et al., 2011; Lee et al., 2016).

## Conclusion

• Parallel, continuous FCM measurements of TCC and ICC at minute resolution with new prototype instrumentation allow for detailed tracking, identification, and quantification of bacterial dynamics at very short time scales.
• Detecting such dynamics shows clear potential for this continuous FCM approach to be further explored as a direct microbial monitor in early warning systems.
• The highly quantitative and temporally resolved measurements open up multiple possibilities for the testing of model-based hypotheses of a myriad of bacterial short-term dynamics from laboratory experiments to full-scale systems (e.g., disinfection, growth, detachment, operational changes).
• Instrument optimizations toward higher temporal resolution, lower delay between sampling and detection, and investigation of other characteristics of bacteria are feasible and should be explored.

## Author Contributions

Experimental design: MB, JS, and FH. Research: MB, JS, RP, BB, GM, and FH. Data analysis: MB, RP, NB, and FH. Writing/editing: MB, JS, RP, BB, GM, NB, and FH.

## Conflict of Interest Statement

The authors declare that the research was conducted in the absence of any commercial or financial relationships that could be construed as a potential conflict of interest.
