# Supplementary material for: Laboratory-Scale Simulation and Real-Time Tracking of a Microbial Contamination Event and Subsequent Shock-Chlorination in Drinking Water
Source: Front Microbiol. 2017 Oct 4;8:1900. doi: 10.3389/fmicb.2017.01900 (PMC5649192; doi:10.3389/fmicb.2017.01900)
Supplement: Supplementary file 1 [file Data_Sheet_1.pdf]

Supplementary information

Laboratory-scale simulation and real-time tracking of a microbial contamination event and subsequent shock-chlorination in drinking water

Michael D. Besmer, Jürg A. Sigrist, Ruben Props, Benjamin Buysschaert, Guannan Mao, and Frederik Hammes

Contents:

1. Further explanations on binning of original flow cytometry data set
2. Advanced fingerprinting of ICC time series

1. Further explanations on binning of original flow cytometry data set

Binning was achieved by counting the number of events with a time tag (given in 10^{-2} s, i.e. 100 milliseconds) in the respective bin (i.e. 1 – 600 x 10^{-2} s for minute 1, 601 – 1,200 x 10^{-2} s for minute 2, etc.). This sum of events within one minute was then divided by the volume analysed within a minute (i.e. 14 µl) to calculate the (average) concentration during this minute.

Table S1: Extract of original data output of continuous flow cytometry featuring single events (represented by black dots in flow cytometric density plots for example in Figure 3) each with a time tag and measurement values for a number of scatter and fluorescence parameters. Time is given in 10^{-2} s (i.e. 100 milliseconds). Multiple events can occur in the same time period of 10^{-2} s.

FSC-A	SSC-A	FL1-A	FL2-A	FL3-A	FL4-A	Time
810	934	6937	649	679	209	165
2150	0	6097	724	148	300	167
0	0	14180	1723	630	95	167
0	254	3587	435	152	143	167
2247	4188	50093	5807	4454	0	167
151	0	3394	454	736	310	168
628	1258	39757	3938	2264	107	168
0	0	6333	1054	700	0	169
0	0	7887	1044	611	103	169
261	294	2881	351	650	159	170
1201	0	7563	1128	705	295	171
0	0	9193	1183	700	171	174
4919	758	29650	3239	1771	198	174
0	213	7055	884	374	186	175
1485	0	3142	691	501	140	179
1347	0	5902	1105	1291	50	179
1796	0	5103	795	242	88	181
0	0	9613	1031	460	28	182
0	461	32937	3486	1738	38	183
272	1211	34501	4148	1819	409	187
0	888	4068	408	94	38	188
6864	824	176920	19468	8781	609	189
0	415	6593	726	508	165	189
1383	44	4518	583	515	125	190
0	0	4754	906	700	134	190
1871	0	7647	1206	769	0	190
1877	1098	54711	5885	2428	11	190
1978	0	8518	1256	615	210	190
1317	291	11516	1507	695	150	193

2. Advanced fingerprinting of ICC data set

The advanced fingerprinting analysis of the flow cytometric data presented in Figure S1 is based on the workflow described in Props et al. (2016). In short, the microbial beta diversity of the intact microbial community was assessed by principal coordinate analysis (PCoA) of the Bray Curtis dissimilarity matrix computed between phenotypic fingerprints. The phenotypic fingerprint for each sample was calculated based on a 128 x 128 binning grid, for four parameters (FL1-H, FL3-H, FSC-H and SSC-H) with a bandwidth of 0.01 for the kernel density estimation. The full analysis workflow can be found here: https://github.com/rprops/Phenoflow_package/wiki/Application:-Drinking-water-disinfection, and we added the data analysis on Github (https://github.com/rprops/RealTime_Cont) and made the data publicly available on FlowRepository (https://flowrepository.org/id/FR-FCM-ZY2V).

As can be seen in the top panel of Figure S1, the fingerprint of the tap water (minutes 1 – 15) was highly stable). Transition from tap water to contaminated water was rapid and reached stable conditions again (minutes 16 – 30) before the beginning of gradual changes in fingerprint during chlorination (minutes 31 – 45). Similarly, the effect of the wash-in of fresh tap water resulted in gradual fingerprint changes (minutes 46 – 80) ending precisely in the initial tap water region of the PCoA. The initial tap water stability, the rapid pollution to a temporary plateau, and the gradual return to initial conditions after wash-in of fresh tap water as indicated by the advanced fingerprinting resembles the flow cytometric intact cell concentration (ICC) measurements. In contrast, the effect of chlorine appears to be more rapid than the contamination in the ICC measurements but only affect the fingerprint gradually. This suggests that chlorination continues to affect the phenotypic properties of the community, long after the effect on the community density (i.e. cell concentration) stabilized. The temporal trajectory further suggests that this is a time-dependent effect, which is in agreement with the nature of chlorination disinfection processes. In summary, advanced fingerprinting can offer highly sensitive descriptions of bacterial dynamics that complement the directly available concentration data.

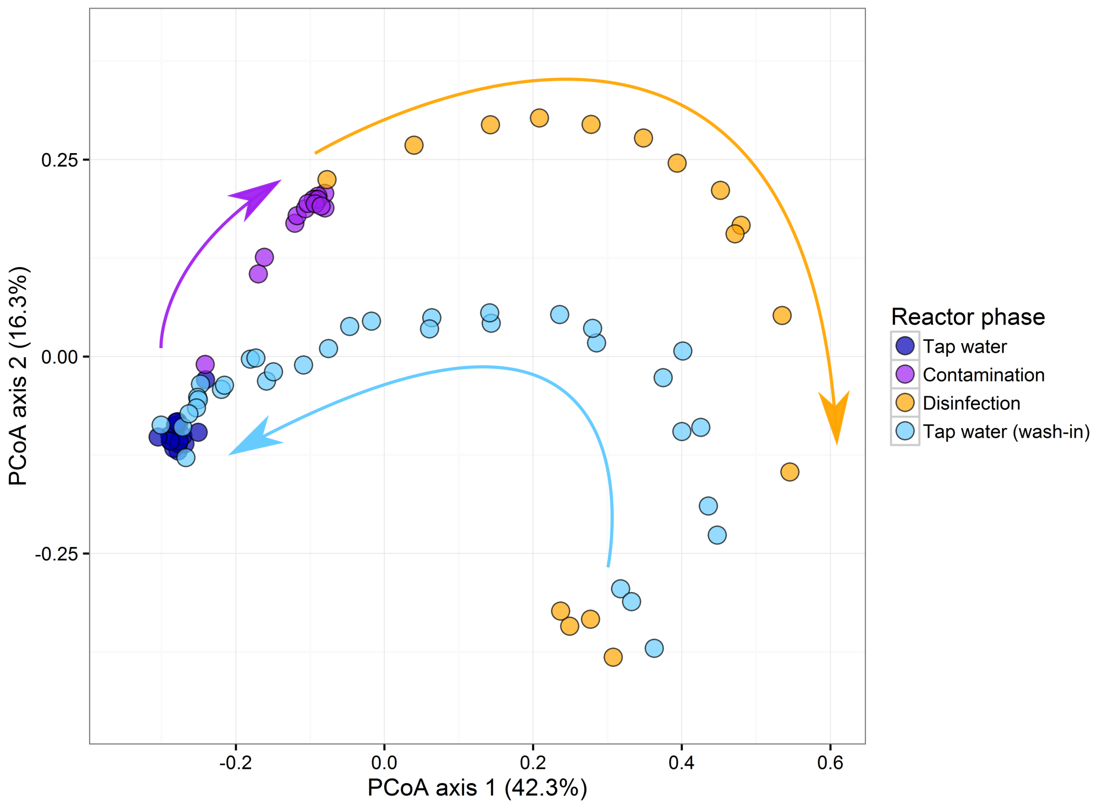

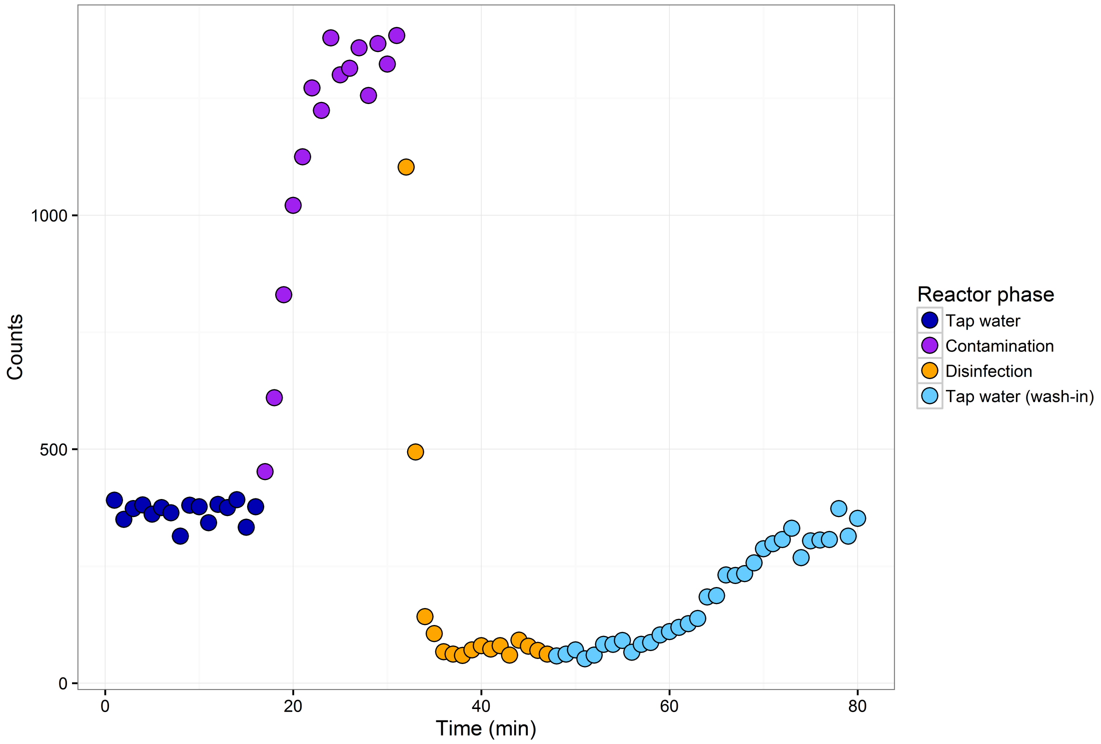

Figure S1: Example of description of changing water quality through advanced fingerprinting based on principle coordinate analysis (top) applied to the time series of intact cell concentrations in Figure 2B (bottom). Colours indicate different phases and arrows temporal evolution of the experiment.
